# Bio-objectifying European bodies: standardisation of biobanks in the Biobanking and Biomolecular Resources Research Infrastructure

**DOI:** 10.1186/s40504-015-0031-1

**Published:** 2015-12-01

**Authors:** Sakari Tamminen

**Affiliations:** Department of Social Research, University of Helsinki, P.O. Box 54, Helsinki, 00014 Finland

**Keywords:** Bio-object, Europe, Infrastructure, Database, MIABIS, BBMRI, Digital

## Abstract

The article traces the genealogy of the Minimum Information About Biobank Data Sharing model, created in the European Biobanking and Biomolecular Resources Research Infrastructure to facilitate collaboration among biobanks and to foster the exchange of biological samples and data. This information model is aimed at the identification of biobanks; unification of databases; and objectification of the information, samples, and related studies – to create a completely new ‘bio-object infrastructure’ within the EU. The paper discusses key challenges in creating a ‘universal’ information model of such a kind, the most important technical translations of European research policy needed for a standardised model for biobank information, and how this model creates new bio-objects. The author claims that this amounts to redefinition of biobanks and technical governance over virtually bio-objectified European populations. It is argued here that old governance models based on the nation-state need radical reconsideration so that we are prepared for a new and changing situation wherein bodies of information that lack organs flow from one database to another with a click of a mouse.

## Introduction

The emergence of new biotechnologies, ranging from techniques and tools directly allowing manipulation of the essential fabric of biological life to expertise in these and their empirical outcomes, has destabilised the way in which the ‘biological’ is understood. It has even brought transformation in how they can be understood. These new biotechnologies emerged mainly in the 20th century – ‘the century of the gene’ (Keller 2000) – and the life sciences’ mandate has since been extended from understanding life to intervening with it (Webster 2012). This extension is in line with developments in other empirically based natural sciences (e.g., Hacking 1983).

Innovation in the ‘bio’ domain increasingly depends on access to abundant biological samples and related information. Since the early 2000s, the life sciences have become dependent on computation power to crunch large datasets and thereby identify and characterise phenomena related to underlying biological mechanisms, pathways, and systems. As biologists ‘think bigger’ than ever before, their work’s appetite for data grows ever more voracious (Hadley 2004). It demands gathering and administration of large collections of samples and related data; however, this is not an endeavour for individual biologists, single projects, or smaller research institutions, nor can it be, on account of the high costs (in time, technological resources, and funding) involved. Biology in itself has become ‘big science’ (Vermeulen et al. 2013), with institutional collaboration and geographically distributed forms of endeavours. Within the big science paradigm, collaboration requires mutually agreed policies, standard operating procedures for sample collection, and common standards for information’s representation and sharing. In other words, collaboration requires a common language and set of core practices, with shared epistemic and ontological commitments underpinning the common research infrastructures now being developed around the globe.

One such major collaborative effort in the life sciences is the Biobanking and Biomolecular Resources Research Infrastructure (BBMRI). The pan-European BBMRI vision emerged from the political recognition that keeping up with developments elsewhere, most notably in the USA, necessitates integrated European research. The development of a pan-European infrastructure is driven by the vision of bringing together geographically dispersed research communities and distinct life-science disciplines (as in biology plus medicine) with the aid of a specific branch of information science fundamentally informing radical transformation in their research practices – bioinformatics (European Strategy Forum on Research Infrastructures 2006, 23).

Large-scale infrastructures are notoriously difficult to build and to manage, and governing them is rife with challenges. Large-scale infrastructure aimed at translating political decisions and statutes into reality is even trickier to implement. Building large-scale infrastructure such as the BBMRI poses a number of challenges for bioinformaticians implementing work toward the political goal, most importantly in the building of a technical platform that could successfully integrate the disparate information models already in use. The integration must link sample collections and studies, deal with natural languages’ barriers and differences in lexicons, and address legal provisions related to protection of privacy.

This article reports on an ethnographic study of the development of the standard BBMRI information model called ‘Minimum Information About Biobank Data Sharing’, or MIABIS (Norlin et al. 2012.). The model is intended to provide a common standard for integrating biobanks across Europe into a common trans-European virtual network of biobanks. It is a working implementation of the proposed biobanks standard, the first attempt to provide an informational backbone for the large-scale biomedical infrastructure platform envisioned by European research policy.

The paper develops two lines of enquiry. Firstly, it contributes to the theoretical debate surrounding the politics of infrastructures and standardisation in the context of information systems (see Bowker & Star 1999; Galloway 2004; Mackenzie 2006; Edwards et al. 2009). In infrastructures and standards, the natural and the cultural interface with and penetrate each other (Mackenzie et al. 2013). They are dimensions along which European biobanks, collections of biological samples, and various social values become mutually entangled in performing of the BBMRI infrastructure. Infrastructures and their standards are a form of technical governance that merits critical examination, since, as Thévenot puts it, they ‘govern life, from living beings to living together in the world. Their extension should give rise to critical reflection on the politics of standards’ (2009, 805).

The paper also offers comment on the recent literature on biobank governance, and the argument is made that, while debates on ethical, legal, and social implications (ELSI 1.0 and 2.0) (see Gottweis & Zatloukal 2007; Kaye & Stranger 2009) are important, more studies of the technical implementation of biobank infrastructure are urgently needed. Digital infrastructures, categories of information, and the related standards shape the way in which biobanks come to life. These, in turn, dictate how tissue samples, data, donors, individual researchers, and research communities ‘meet each other’ (Bowker & Star 1999), along with how they can be governed (and when they cannot). At the same time, scientific and technical developments in the biosciences are driving the integration of geographically remote biobanks and their data, promoting openness and data-sharing under what is otherwise known as the ‘big data’ paradigm. Here, creation of common standards and the existence of biomedical infrastructure are key requirements for collaboration across time and space.

This is also why, today, ‘biobank governance is also increasingly characterized by its deterritorialized, transnational/global character’ (Gottweis & Zatloukal 2007, 210). Yet there are no internationally binding regulations addressing such activity. Accordingly, this paper and, specifically, its discussion of MIABIS also elaborate on the claim that ‘our current governance system for research is unable to provide all of the oversight and accountability mechanisms that are required for this new way of doing research that is based upon flows of data across international borders’ (Kaye 2011, 77). Interestingly, one of the historical roots of MIABIS – the information standard whose examination constitutes the heart of this article – lies in the Swedish Data Inspection Board’s prohibition of linking national biobank data between separate banks and registries. That restriction prompted the researchers involved to devise a novel approach to large-scale biobank infrastructure and standards development, one involving technical measures to overcome strict privacy-regulation statutes, both nationally and EU-wide.

In this piece, then, I try to illustrate how the ‘political’ in biobank infrastructure’s development is related to different meanings of ‘politics’ or ‘the political’ within biomedical informatics and its governance. By analysing the development of MIABIS, we can see how European research policy has become translated into four distinct areas of infrastructure growth, all governed predominantly by means of *ad hoc* technical standards. I start by asking how biobanks and the biological samples they hold manifest themselves in research policy. Next, I explore how European biobanks are technically aligned and re-articulated as a networked gateway to samples and information from the studies for which those samples were collected, along with how all of this information becomes standardised. I then describe how said technical articulation is institutionalised and managed within the MIABIS working group. Finally, I discuss how the MIABIS standard construes the developing ‘bio-ontology’ of Europe’s biobanks.

I seek to illustrate how the MIABIS standardisation process demands technical re-articulation of the definition of biobanks and, in line with their informational, technical, and biological nature, what kinds of bio-objects they embody to meet the infrastructure-policy goals set. The creation of an information standard shifts the boundaries of the private and public between individual donors, biological samples, and data in their becoming visible – to the extent specified by the MIABIS standard – to users of BBMRI-related infrastructure services, researchers, and research communities alike. This renders the process of networked, international virtualisation of bio-objects immune to existing national-level privacy regulations pertaining to biobanks. In this way, the technical is also political.

For the analysis, I examined empirical material drawn from multi-site ethnography and mixed methods, including BBMRI infrastructure-policy documents’ analysis, review of scientific publications and grey literature related to MIABIS, ethnography from the HandsOn: BioBanks 2013 conference and its workshops dealing with biobanks’ standardisation, and interviews with 12 key individuals involved in MIABIS development in 2013–2014. Four of these key persons working on BBMRI development and the MIABIS model were interviewed several times in the course of the research. The interview material was thematically coded, analysed, categorised, and interpreted, with all interview output contextualised with ethnographic observations of the discourse and practice of the BBMRI and MIABIS projects.

## The political anatomy of biomolecular research infrastructure: articulating the BBMRI vision and challenges

The concept of creating pan-European research infrastructures has long been in the making. Its first political articulation was seen in the 2006 roadmap report of the European Strategy Forum on Research Infrastructures, commissioned by the Council of Europe (European Strategy Forum on Research Infrastructures 2006). The report identified 36 key fields of research in Europe exhibiting a need for large-scale infrastructure and, in this connection, mooted a pan-European research infrastructure for European biobanking and biomolecular resources as of high priority. The authors recognised that the life sciences have undergone a profound transformation, wherein ‘[b]iology (including medicine) has become an information science. Modern science is inconceivable without recourse to well structured, continuously upgraded, massively enriched at exponential rate and freely accessible databases’ (*ibid*., 23). Further, as biology becomes ever more an information science dependent on large-scale datasets, the integration of bioinformation databases has become a top priority in life-science research. This policy priority effectively underpinned the political framing of the BBMRI structures.

The technical and practical prerequisite of integrated large-scale databases was reflected in the 2008 European Science Foundation report on population surveys and biobanking. This explains:

[G]ood, inter-operable Information Technology (IT) systems are required so that information contained in the different datasets can be adequately mined by integration or, at least, interfacing, and efficiently linked to relevant information from other sources. The fact that many biobanks or biobanking networks use different IT platforms and different message formats and terminologies represents a significant obstacle to communication with, within, and between biobanks. (European Science Foundation 2008, 8)

In the BBMRI context, then, biobanks translate first and foremost into networked databases embodying the political vision of Europe-wide research integration, with the promise of new scientific breakthroughs in the biomedical arena and economic innovations. Starting with a three-year Preparatory Phase in 2008, it grew into a 54-member consortium. It is widely considered to have been successful. In its key outcome, it defined the concept of a distributed pan-European biobank infrastructure, which was implemented legally under the European Research Infrastructure Consortium (BBMRI-ERIC) entity in early 2014. It became the first EU-wide infrastructure envisioned in the research-policy reports referred to above, funded by the European Commission.

The aim with the BBMRI is to provide easier digital access to and more efficient use of the high-quality collections of biological samples of human origin, now scattered across individual biobanks throughout the EU. The idea of the BBMRI is based on the political vision of creating a virtual network that acts as a ‘gateway’ (the metaphor used in the tagline in the official BBMRI logotype is ‘gateway for health’) to biobanks all over Europe and serves as the key platform for European biomedical research. It is aimed also at facilitating collaboration with pharmaceutical companies and linking other stakeholder communities to the infrastructure’s development.

The value of the BBMRI is spelt out in the description of the BBMRI’s concept – the 'Business Plan' (Zatloukal, Vuorio & Dagher 2012) – which explains how at the level of political discourse, the BBMRI is represented as one coherent platform, an idea based on envisioned generative potential for networked research and opportunities for collaboration. At practical level, however, it is not one entity, as it is made up of a network of relations, national hubs for member country biobanks, and human biological samples, all connected through standardised ways of representing, aggregating, and communicating biobank information.

Thus, for the political vision to become reality, the development of the biobank infrastructure required, as it still does, continuous articulation work (Strauss 1985; Bowker & Star 1999) encompassing diverse biomedical databases and related lexicons, local categories of biomedical information, and distinct standard operation procedures. Most importantly, for a logical starting point the BBMRI project needed to specify a well-defined architecture for the biobank network and within that also the information model for the banks holding biological samples and related data. Only a well-defined architecture and a model allowing good circulation of information can bring about development of a one-stop-shop digital point of access to biological data all over Europe.

This challenge is reflected in these comments in the summary section of the final report from the Preparatory Phase submitted to the European Commission:

Another great technical advantage of the PP scheme was the concept towards a common IT- infrastructure of BBMRI-ERIC, which will consist of a network using the hub and spoke topology to connect the different National Nodes, which are geographically spread through Europe. The planned IT-infrastructure employing a federated database architecture will integrate the complex network of hubs, members and associated partners in the Implementation Phase of the BBMRI. (Zatloukal 2011, 5)

The architecture for the infrastructure as explained in the final report, which later became accepted as the key model for the digital structure of BBMRI-ERIC, is simple in theory but complex to build in practice.

The simplicity of the architecture lies in the delegation of primary responsibility for information-generation and communication to ‘National Nodes’. No attempt is made to establish a central information repository that would be the ‘platform’, a central BBMRI biobank. Instead, each National Node has a member-state-nominated director acting as the national co-ordinator, with a seat on the BBMRI Management Committee. These co-ordinators are tasked with the identification and co-ordination of the national biobanks and biomolecular resources (e.g., those of the relevant country’s universities, hospitals, research institutes, and resource centres) and with forming a coherent view of local activities and entities. The role is rooted in co-ordination at national level and mediation between local or regional biobanks and the BBMRI network. However, National Nodes need not have a legal form or any legal control over the biobanks and their samples. As their task is to identify and list biobanks at national level and then report back to the BBMRI network, they do not need any legal mandate. Instead of legal accountability, the scientists and developers of MIABIS have pragmatic techno-political accountability for translating EU research policies into practice.

In addition to the distribution of responsibilities, the ‘hub and spokes’ topology makes the infrastructure modular and scalable both horizontally (between countries) and vertically (within the countries’ various regional and local medical communities). However, scaling of the network across research communities and the EU itself remains a practical challenge. How can a national co-ordinator who lacks a legal mandate get very different medical communities enrolled in the infrastructure? Why would they join? After all, they have laboriously collected the samples, run studies, and constructed their biobanks from scratch. The concern is greater still if the individual institutions have no say in the governance, how the data are used, and who is granted access. Also, they may not even be assured of the right to share the collections and data gathered. (Cf. Meijer et al. 2012)

**Fig. 1:**
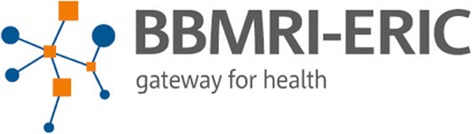
The BBMRI logotype

A second concern, following on from this, is that the distributed network infrastructure becomes directly dependent on minute technical details and the implementation of the model for sharing of biobank data. The sharing model becomes an underlying technical rationale and a distributed digital condition for the creation of a virtual network (Fig. 1).

Furthermore, for the infrastructure to function in real life, each node in the BBMRI network must have mapped the new information-sharing model onto its biobanks’ current systems for representation of samples and related bioinformation. The infrastructure crucially depends on an information model that renders it possible for the ‘spokes’ to connect the BBMRI nodes. It should be clarified here that, instead of denoting actual physical connection, ‘spoke’ is a metaphor for the technical existence of meta-biodata exchange. Spokes’ shape and form follow from conditions for the format of the information flows between nodes. These are set in the biobank information representation standard.

The above-mentioned challenges were recognised in the Periodic Report summary from the Preparatory Phase, wherein major future challenges to implementation and development of the BBMRI were explained in detail. They were articulated in terms of ‘divergent views’ among participating scientists and officials with regard to governance; funding and representation rights related to the data gathered; varying ownership details and access protocols for samples and biodata; and heterogeneity in the formats and languages used to capture and store molecular, clinical, and lifestyle information (Mayerhofer 2011). Clearly, the challenges were well understood by the scientists in the management team for the BBMRI Preparatory Phase. One of them, a senior scientist, explained the challenges to me in detail, employing more vivid empirical examples of what the integration challenges meant on the ground:

On the one end, we have pathologists who have their own special *ad hoc* collections that could be very valuable for research purposes if combined with larger datasets. But many of them see the collections as their personal property, with little to no incentive to give anyone access to what they see as theirs. One can argue whatever about their rights to the samples and so on, but the reality [is that] you won’t get them enrolled if you don’t give something in return. (Interview from 13 December 2013)

The possibility of divergence in collaboration- and data-exchange-related views between medical professions may be obvious from the above quote. Here, pathologists were cited as one group of professionals with potentially valuable collections. They were seen as a group that is difficult to convince to join the BBMRI effort because of questions related to data ownership and lack of immediate return on their investment. Both divergence of views and the lack of a common vision and benefit policy play a large role in the platform-building. A few moments after voicing the views above, the senior scientist quoted continued by identifying easy starting points and some medical communities that are much more willing to take up the BBMRI platform:

[A]nd at the same time we have epidemiologists who work with large population samples and who are an easier group of people to work [with]. For many projects, the funding bodies already require that the scientists place the samples collected within a study in a recognised biobank for potential reuse. There are major differences between researchers, institutions, and their policies with regard to sample- and data-sharing, which poses major challenges [if we wish] to get everyone on board [with] this vision of the common infrastructure. (Interview from 13 December 2013)

Though the challenges identified above are real ones, various developments, ranging from new conditions for funding to particularities of certain types of medical research, render the first steps and building of the infrastructure project’s first spokes much easier. Nonetheless, the interviews reveal considerable continuing discrepancy between the challenges identified in the official BBMRI documents and the practical complications in infrastructure-building. While in the official documents the practical business of building a common research infrastructure has been framed mostly as a ‘technical problem’ of database interoperability and integration, a challenge thrown out in the policy documents to the bioinformaticians, the definition of a common information model was called fundamentally into question. The teams working on the model recognised how difficult the creation of a single standard was, for there was no ready agreement upon a common vision by all stakeholders. The challenge was articulated simultaneously at many levels, from misgivings of individuals and profession-specific entities to a policy problem couched in cultural contexts of the diverse medical communities in the European Union. The challenge is nothing less than that of finding ‘European common ground’, agreeing on shared ground rules, articulating the abstract idea of Europe in dealings with specific countries and institutions, and linking the infrastructure policy with the everyday praxis of medical communities and individual researchers.

## Mapping of the network: the BBMRI and European biobanks

The political idea of European unification has a long history that has always revolved around three questions: what it is exactly that needs to be unified in Europe (economy, politics, institutions, etc.); how the unification could take place; and, after unification, the question of the official language of Europe and its relation to the diversity of languages found in the Member States (Kraus 2010). However remote these political problems may at first seem from the context of the life sciences and their medical-database infrastructure, these three challenges are ubiquitous also in the practical labours aimed at defining the BBMRI information models for biobank integration.

The life sciences strive toward universally generalisable knowledge and for representation in uniform ways. The digital world, in turn, could not function without ‘universal’ statements, the precise codification of information categories, and well-tailored search algorithms. But neither scientific language nor the knowledge produced by science standardises itself. Instead, standards have to be socially negotiated and decided upon actively at the level of both natural language and digital representation, as a large body of research in science and technology studies (STS) has demonstrated (e.g., Timmermans & Berg 1997; Berg & Timmermans 2000; Edwards et al. 2009).

From early on, bioinformaticians had to face the three above-mentioned challenges inherent to unification in the context of ‘European biobanks’. Accordingly, when the BBMRI Preparatory Phase was launched, the key questions and tasks for Work Package 5 (WP5) reflected the political challenges attendant to integration across Europe. What kinds of biobanks exist within the member countries? Where are they located? How can definitions commonly agreed upon that pertain to biobanks and their data be devised when diverse means of definition exist? What is the best way to integrate data about them and the bio-objects they hold? (For discussion, see Zatloukal 2011, 10–11).

**Fig. 2:**
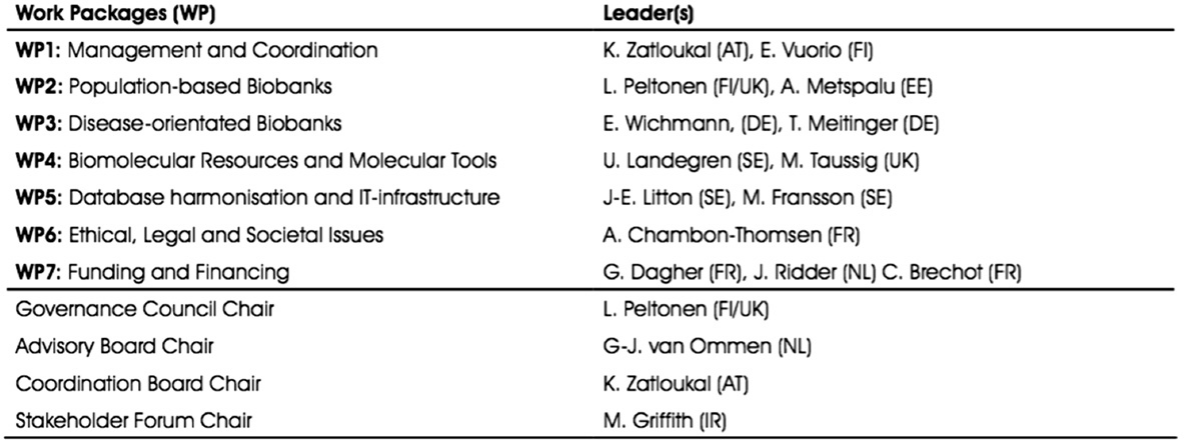
BBMRI preparation-phase work packages (WPs) (from Salminen-Mankonen et al. 2009, 5)

The team responsible for WP5, ‘Database Harmonization and IT-Infrastructure’, was led by a bioinformatics team from the Department of Medical Epidemiology and Biostatistics at Sweden’s Karolinska Institutet. For this team, the challenge of biobank infrastructure and database harmonisation was never primarily technical. Rather, the first real problem was the mapping of European biobanks in the unclear situation described in the Periodic Report summary extract above (Fig. 2).

One of the first tasks in the preparation phase was to draw up a map of Europe’s biobanks. Work began to inventory existing population-based and clinical biobanks via a questionnaire-based survey. The core questionnaire was sent to initial participants in the Preparatory Phase and was mandatory for those who wished to be registered in the BBMRI. In addition to biobanks’ and samples’ identification, the questionnaire probed the thoughts of the respondents on numerous other issues, such as standardisation of study procedures, collection and handling of data, IT solutions, and matters related to legal and ethical issues and to funding (Salminen-Mankonen et al. 2009).

**Fig. 3:**
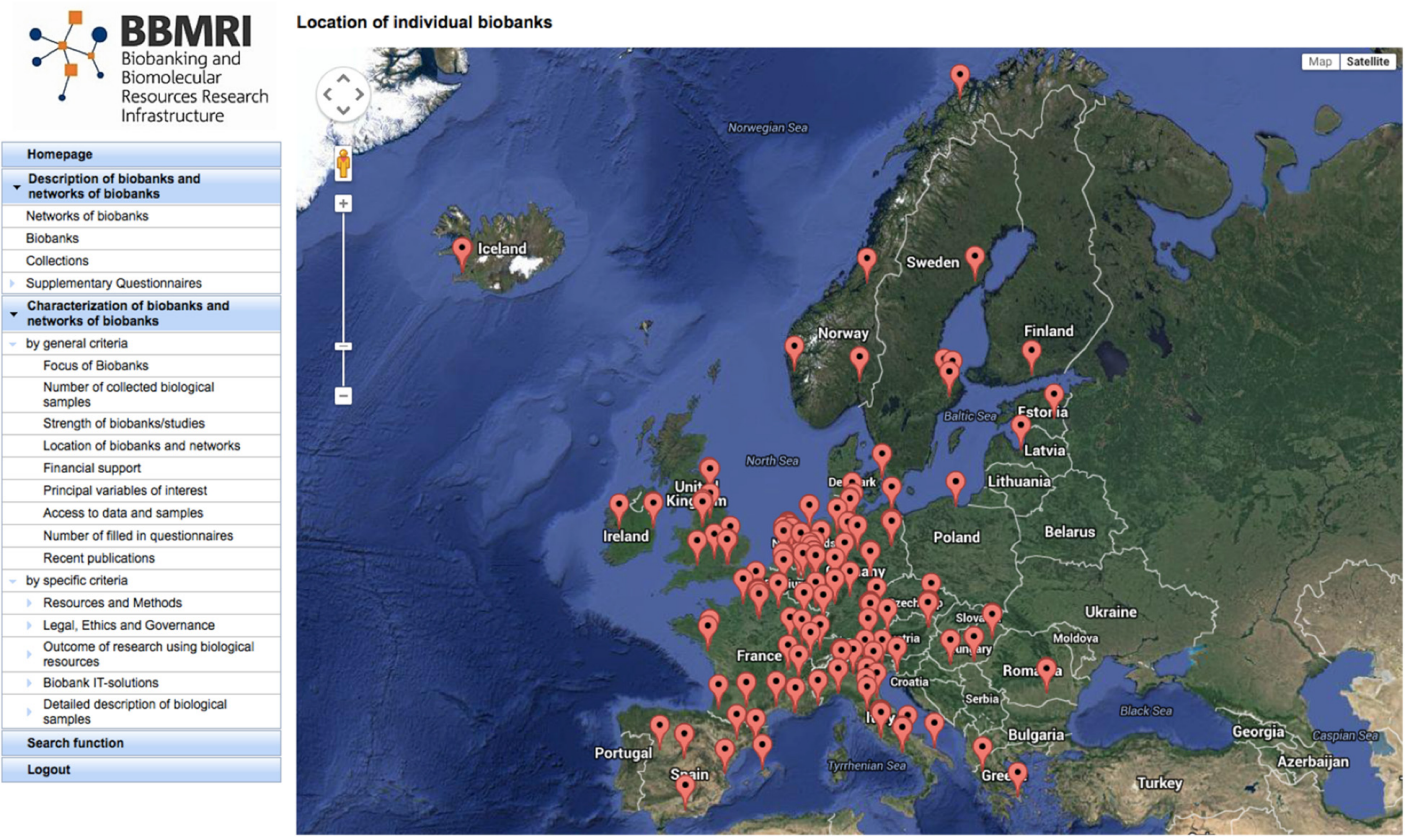
The BBMRI biobank catalogue

In all, across the EU, 315 biobanks were found and registered, accounting for, in total, just over 20 million samples derived from humans (blood, cell lines, serum, tissue, etc.), more than a tenth of which are DNA samples. The outcome of the survey was used as the basis for developing the BBMRI biobank catalogue, which now hosts and represents information from the component biobanks online^1^ (Fig. 3).

When interviewed in 2013, a senior figure from the Karolinska Institutet team explained why the catalogue is important as a first step towards European biobank integration:

Technical challenges are not the issue. Five years ago maybe, people talked about the software and so on. But not anymore. It’s like cell phones: people don’t care about the operating system but instead want services or apps that work. So what we tried to do in the catalogue was that even if countries have their own way of doing things at local level, they could still upload information of the biobank to the catalogue. (Interview from 21 November 2013)

Thus, even with strong differences remaining among European countries’ medical communities, their biobanks, and ways of representing sample collections, the survey and online catalogue provided one way to standardise the information, by generating it *de novo*. This proved a time-consuming way to gather biobank information in the digital era. Even if the information was in a standard format, the work was labour-intensive. The ‘digital message format’ for the information gathered followed a structured 14-page template from the questionnaire, later translated into online form. This standard is far from ideal, dependent as it is on factors such as the biobank representatives’ will to do extra reporting ‘on top of the normal work’ (even though filling in the survey form was mandatory for joining the project). It was vulnerable also to issues of the representation format being difficult to share, being impossible to ‘enforce’, and leaving room for local idiosyncrasies to emerge – various groups could fill in the form differently. Also, availability of this kind of biobank information does not render the digital biobank databases themselves interoperable. It only facilitates making biobanks across Europe discoverable.

**Fig. 4:**
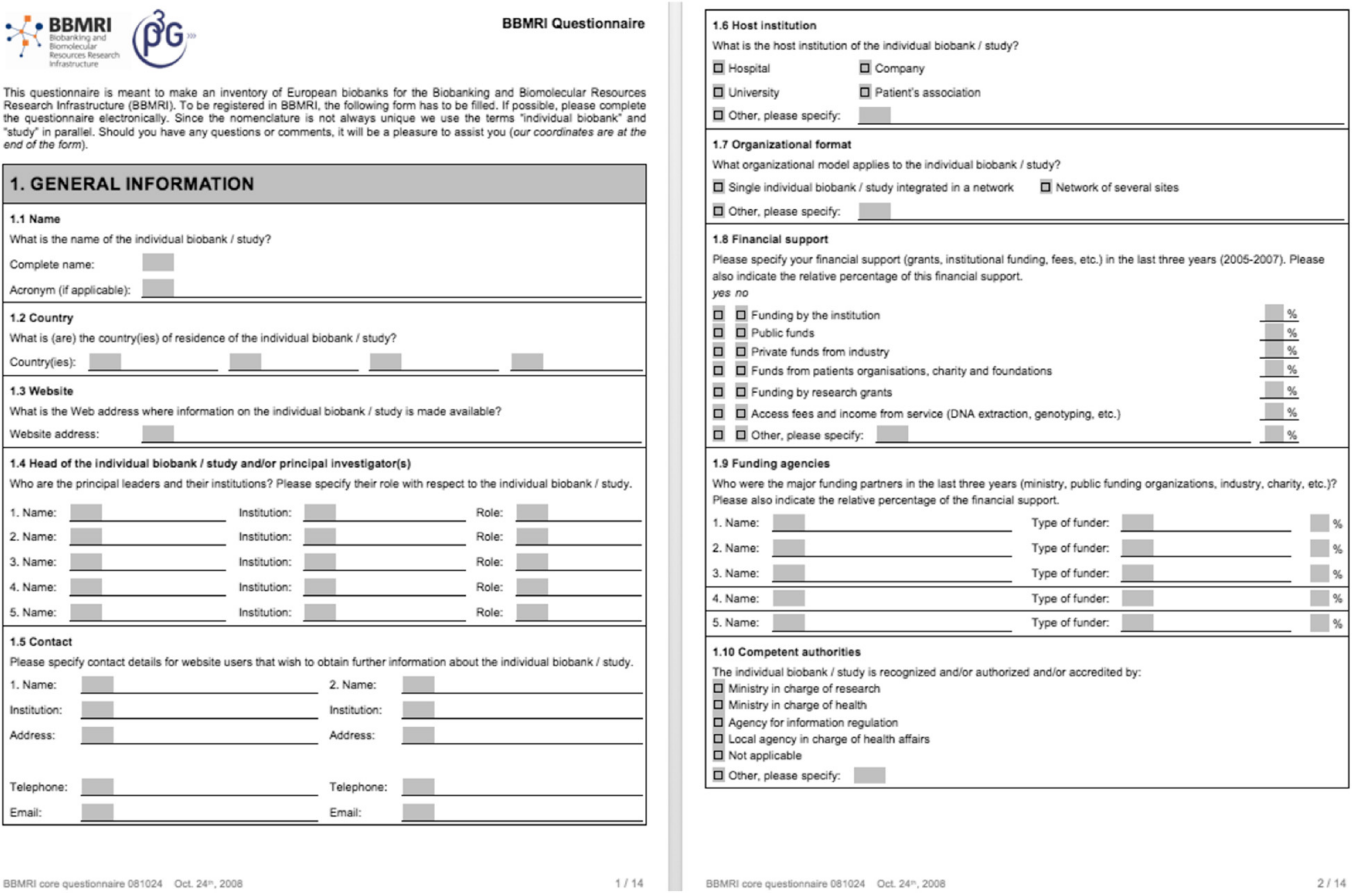
The first two pages of the original BBMRI biobank survey standard document

The online biobank catalogue as described above goes some way to addressing the first problem in translating the European research policy vision into an infrastructure. Most importantly, it answers the empirical question of what the European biobanks are, where they are, how many exist, and which are to be listed and incorporated under the BBMRI scheme. Even in this basic initial form, it opens the possibility of searching for various sample types, organs, and ICD-10 disease groups (with a dozen filtering criteria) across European biobanks, and the result list can be narrowed by physical location. But the question of how the samples and data stored in biobanks could be integrated remained open (Fig. 4).

## Developing the European language of biobanks

Why is a well-defined biobank dataset beyond the one provided by the BBMRI catalogue so important for digital research infrastructure development? The problem of having only simple discoverability (of biobanks, sample types, and studies) is that the scheme operates at too high a level of abstraction to contribute effectively to rapidly paced research. Furthermore, the information about biobank collections has not been coherent across all entries. All communication – especially in a multicultural environment such as Europe – needs a well-defined language if it is to unfold in such a way that the conversations among parties make sense to all participants. This requires a shared lexicon and semantic clarity related to biobanks: to bridge the gaps between the 24 official languages found in the EU, precise translation between these languages becomes a necessity. Indeed, in pursuit of this end, the European Union has set up the Directorate-General for Translation (see also Rogers 2007).

**Fig. 5:**
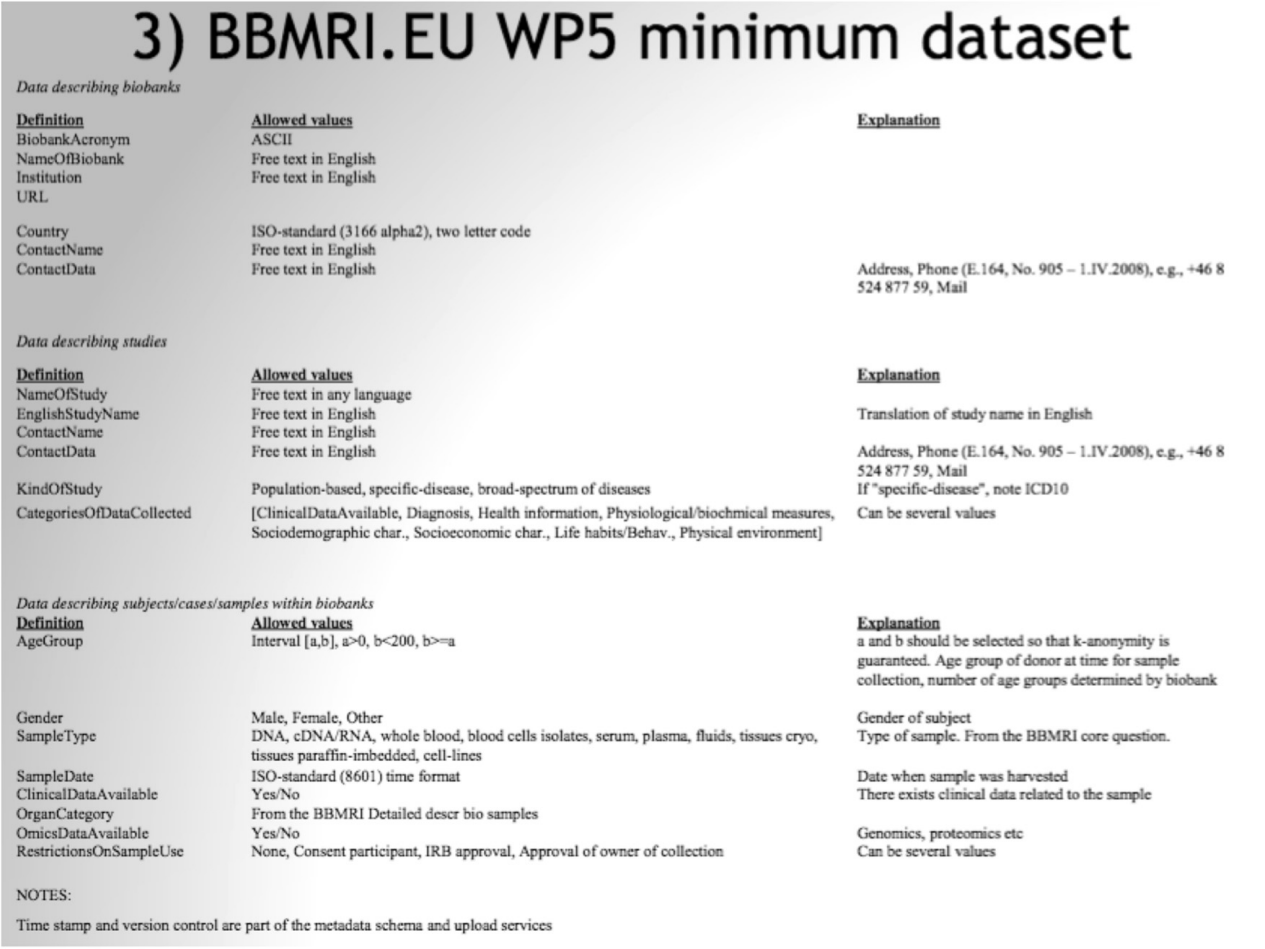
The initial minimum dataset – a screenshot from a bbmri.eu WP5 PowerPoint presentation

More specifically, generating a standard information model in the context of technical biobanking standardisation efforts demands, as prerequisites for integration, explicit definition of a limited set of semantic entities, enumeration of their attributes, and systematic mapping between databases in line with the model. This is why a precise language for talking about key concepts related to biobanking was explored and an initial ‘minimum information set’ common to all biobanks was suggested at the end of the Preparatory Phase. It included 54 ‘entities’ of data, ranging from biobank definitions to suggestions as to how to represent data on research subjects (see Fig. 5).

Defining the core units of bioinformation and their mutual relationships is similar to problems faced in natural-language communication in multicultural environments. There is a need for clear communication whether one is asking directions as a tourist in an unfamiliar city or establishing a common working language in a professional setting. Understanding the topic of the discourse and the meaning of the basic utterances is achieved within evolving interaction, with interaction-repair sequences and a constantly developing relationship encompassing a sense of shared history. Not even the most basic category – the concept of the biobank – escapes politics of language in the information-modelling and database-integration work. The head of the Swedish bioinformatics group explained the politics of language, standardisation, and the relationship of these to the challenge of biobank information’s standardisation thus:

One of the issues in Europe is language. You see, the terms and definitions you use for biobanks are a sort of a problem because there are so many different borders with BBMRI and everyone [has] their own terminology that differs from each other. So we have created a lexicon. The current version default [for the database information standard] is English, but what we did was to provide the information and the service in 10 different languages. (Interview from 21 November 2013)

If the concept of biobank in each EU language denotes a specific local, country-level configuration of sample collections, data, and study information, how can a scheme make sense of them all? The Swedish bioinformatics group explicitly anticipated the issue of terminology and created a lexicon of biobank-related concepts, providing its own translation of these between 10 European languages. Thus, standardisation between languages was deemed the first translation required. However, this standardisation can only occur within the BBMRI context. The definition of biobanks is not only a matter of providing inter-language equivalence for the EU’s various medical communities. This is because the concept of biobank can differ in content also within the various epistemic communities within a particular member country. The medical community may have one working definition of ‘biobank’ while the legal community might entertain another. This is how the head of the team unpacked the elements at issue:

[A]t the same time, when you look at the legal challenges there, we have different legal definitions for biobanks and all things related to them at national level. So we have two different baskets of challenges. The definition of a biobank differs between languages. What is a biobank? Europe does not speak only English. Many adopt the English term, but for many countries the definition of a biobank is hard to find or come by in their language. Because the definition in [that] native language does not exist. This means there cannot be any legal regulation over biobanks within that country. Yet if biobanks are regulated, there might still exist a different idea of a biobank within [the] legal and medical communities. It’s no use to discuss laws if [the] medical community talks about one thing and the lawyers about something else. That is another key issue. (Interview from 13 December 2013)

**Fig. 6:**
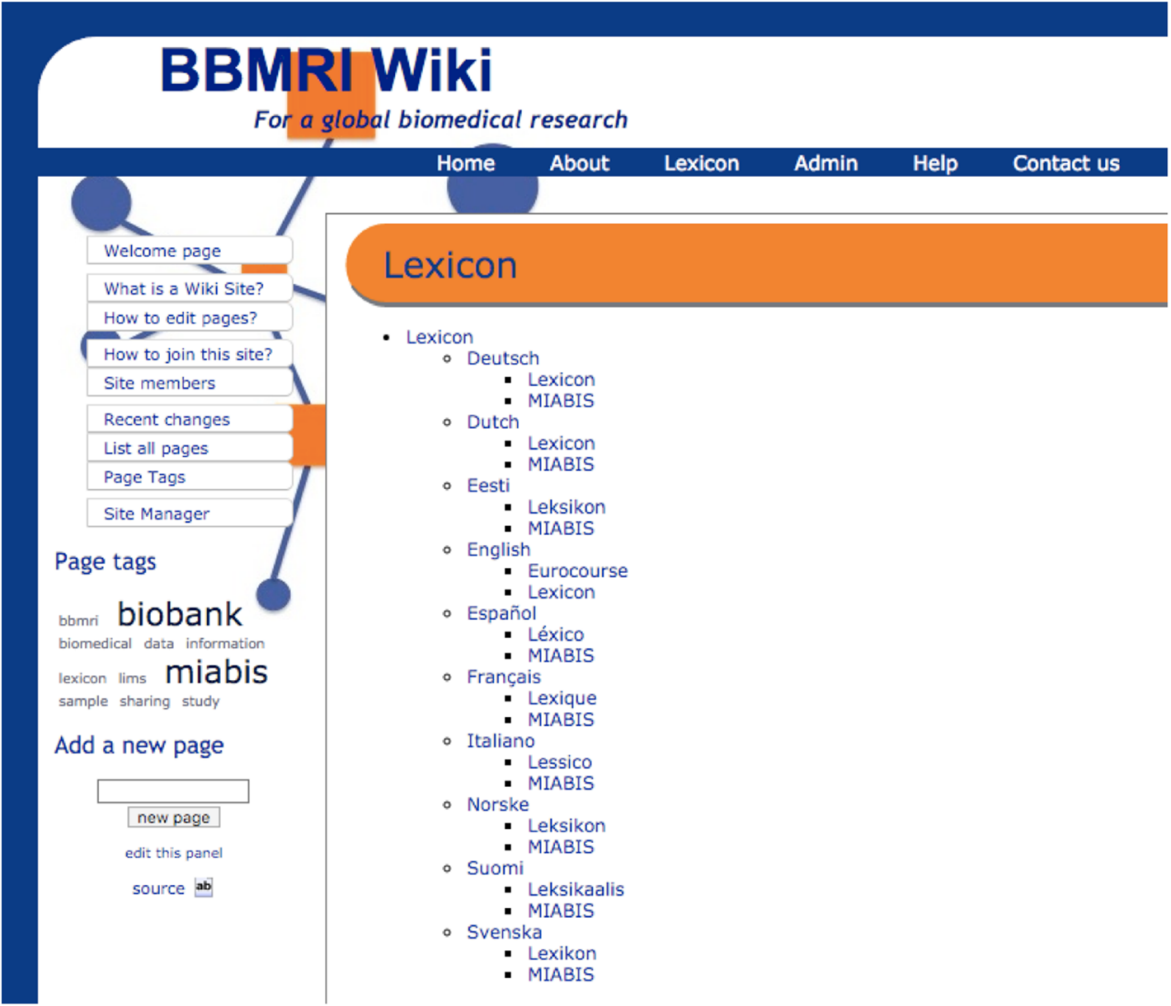
Screenshot of the English lexicon developed by the bioinformatics group

The two ‘baskets’ of challenges, definition of biobanks in different languages and the legal challenges, are thus interconnected in work to develop a Europe-wide information model. Standardisation efforts’ adjustment of all language related to biobanks and the corresponding set of concepts surrounding them can provide stability for the standard. In addition, this is why various national representatives in BBMRI work need to adopt something approaching the same fixed language of European biobanks, with a well-defined biobank lexicon. Doing so is crucial for achieving ‘crystallization of the standard’ (Timmermans & Berg 1997, 295) (Fig. 6).

Most importantly, the Swedish group’s lexicon specifies not only what a biobank is but also what key categories of information are common to all biobanks within the BBMRI. For the first version of the standard (version 1.0), ‘biobank’ was defined as ‘a collection of biological material stored, together with the information attached to the material, for one or several purposes’.^2^ This was updated to be more specific only with version 2.0, in 2014. Aware that the BBMRI definition of biobanks might not match the legal definitions in all EU countries, the team at that time added a remark about the legal status of the definition. The official wiki page on the standard reads thus:

We choose to present two definitions for the term ‘Biobank’. One by BBMRI-ERIC and one by P3G [the Public Population Project in Genomics and Society]

[…]

1. ‘Collections, repositories and distribution centres of all types of human biological samples, such as blood, tissues, cells or DNA and/or related data such as associated clinical and research data, as well as biomolecular resources, including model- and microorganisms that might contribute to the understanding of the physiology and diseases of humans’ (BBMRI-ERIC)
2. ‘An organized collection of human biological material and associated information stored for one or more research purposes’ (P3G)

It should be clear from the interview comments above that those working with the MIABIS definition of ‘biobank’ were well aware that some countries do not have a legal definition for biobanks. Yet the group needed a ‘technical’ definition, which they derived from two well-known sources in their field. No problem had to follow if there was no relationship between this technical definition and the national legal definitions already in place in some European countries, and it could introduce a definition for those countries lacking one.

The solution entailed concluding that the technical and legal definitions may differ and producing a technical (medical) definition without a direct link to the various legal ones. The legal questions and challenges are thereby bypassed, and a technical standard lexicon provides the *ad hoc* definition to which all parties to the BBMRI must conform. This is a technically informed strategic move in the definition of biobanks at the European level and, as such, raises a question about the right to define such a thing. The BBMRI, the digital model, and the final form of the information itself depend on this: on whose terms is the final standardisation going to be defined at the European level?

## Privacy and workaround policy: the roots of the MIABIS model

Clearly, therefore, the definition of biobanks and the whole language surrounding them (exemplified here by the lexicon) are not only technical but also inherently political. In fact, the entire idea of the data model for biobanks is connected at a very deep level to the tug-of-war between medical research interests and personal data protection laws. While research communities attempt to create a European ‘free-trade zone’ for biobank information, legal scholars and data protection boards attempt to balance rights to privacy against the potential benefits of new digital technologies capable of aggregating both individual- and population-level data.

This tug-of-war becomes evident when one digs more deeply into the history of the idea of MIABIS, the powerful notion behind the standardisation efforts aimed at providing the common information model for European database integration. In rewinding through the history of the MIABIS information standard, one ends up finding a direct confrontation of EU research policy, the needs of the biomedical research community, and issues of personal privacy. When interviewed in August 2014, the individual responsible for MIABIS co-ordination recounted a key wrinkle in the birth of the idea, situating the story explicitly in a confrontation between developers of novel biomedical platforms and the data inspection board of Sweden:

What happened is that we wanted to extend the Swedish Biobank Registry with sample data for researchers, to make it easier for researchers to find samples and related biobank data, but the Swedish Data Inspection Board said that we couldn’t do it; with individual[−level] data for research purposes, due to a variety of reasons, we needed explicit consent. Because we were not able to provide it, we created a searchable database with only metadata about sample collections, which do not violate the privacy of the donors. You could say that it was the Swedish Data Inspection Board’s prohibition that pushed us toward the new ideas of using not the data of donors but aggregate data on the level of the biobank and related metadata. This, together with a dataset developed during the BBMRI preparatory phase in 2008 and 2009, paved the foundation for MIABIS. (Interview from 28 August 2013)

The prohibition of using medical registry data as part of the Swedish biobank sparked technical imagination and resistance. The researchers came up with a ‘workaround policy’ and an associated technique for handling biomedical information. The power of the Swedish Data Inspection Board generated a new, and unregulated, possibility for bioinformatics and data on patients, samples, and studies.

Here we could extend the words of Foucault, who once identified the creative power of resistance generated by prohibitions and repression, to the context of biodata. While the Data Inspection Board demanded particular asceticism with regard to aggregating biobank data, what emerged in response to the prohibition did not display data asceticism at all. Instead, and on the contrary, an intensification of the data on the body, with problematisation of health and its operational terms, ensued (cf. Foucault 1979, 122–123). This applied a logic of biopower and governance that pools and aggregates data on populations of choice. It is a governance logic typical of modern power, one wherein individuals are valuable insofar as they are recorded and can be seen and identified as part of a population-level aggregate. It also represents a new way of managing biobanks, wherein the aim no longer is to bank large numbers of people for one purpose, for one-time use, and for one study. Instead, the objective is to provide a totally new way of doing research, one that is at its core ‘networked’, not local or nationally bound.

The generative resistance in relation to biobank regulation and the Data Inspection Board was successful. As with the question of ‘sex’ in the Victorian era, addressed by Foucault (*ibid*.), the idea of linking biobanks found a new manner of expression, now in mutated form. Again, instead of directly using medical-registry data on patients and their samples, the biobanks started using aggregated data, thereby intensifying the data on life and health. These involve not one biobank, sample collection, or study but several at once.

Today, MIABIS covers three core datasets, describing biobanks, sample collections, and studies, complete with 37 attributes that define all of these in detail and in a manner that enables implementation via a general standard for integration of Europe’s biobank databases. This information model is centred on ‘metadata’ that include details of biobanks, the sample collections they hold, and studies performed on the sample collections at an aggregate level. Thereby, the model escapes the restrictions of legal provisions on personal data protection imposed at national and European level.

**Fig. 7:**
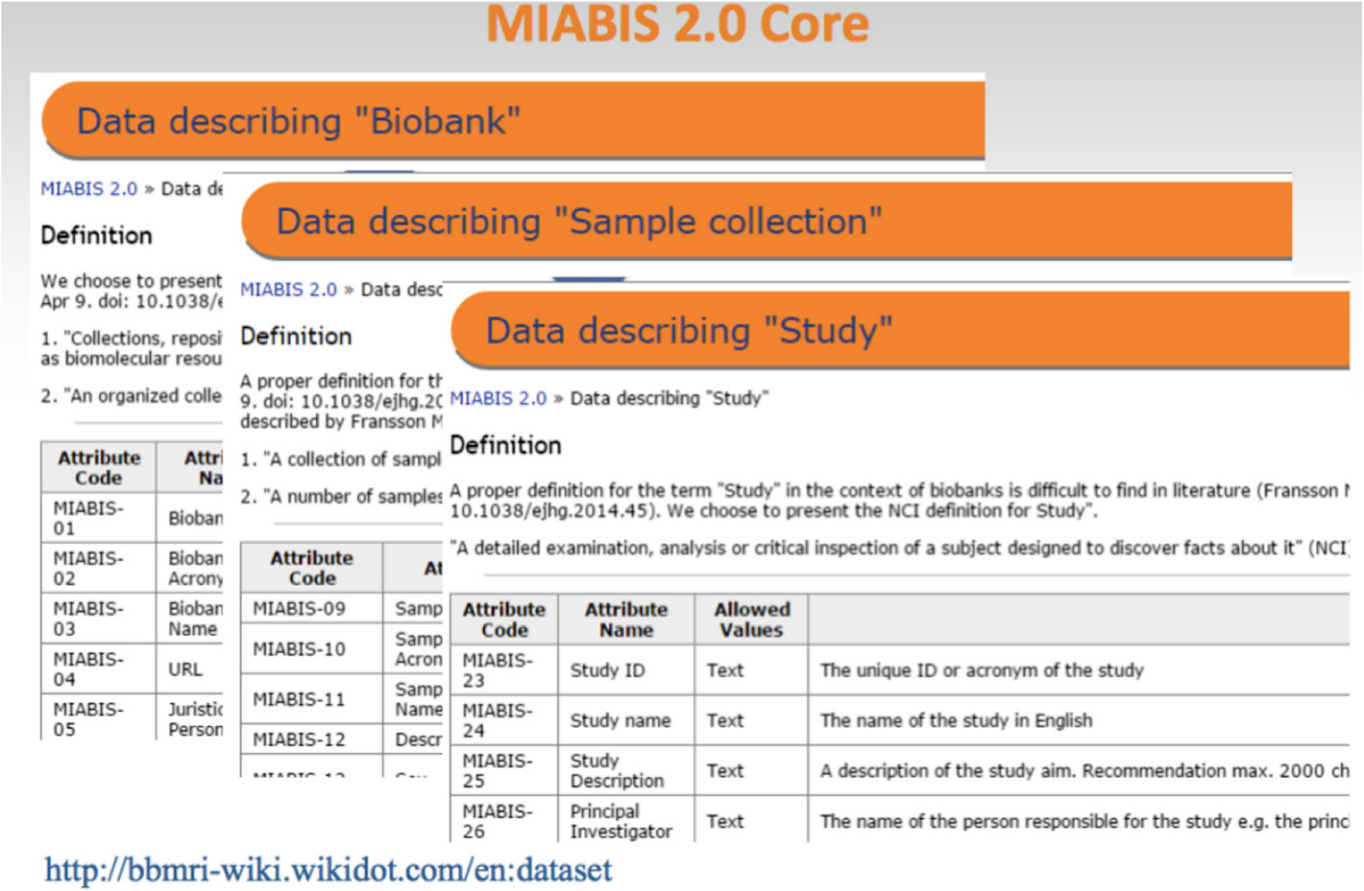
The MIABIS 2.0 information model with its three core datasets: data describing biobanks, sample collections, and studies

Although not yet ‘ontologised’ (organisationally frozen as the stable information model), it still stands as the clearest, simplest, and most ready-to-implement model for integrating European biobanks’ databases. The BBMRI European biobank catalogue is to be restructured in accordance with the MIABIS structure. National hubs are expected to implement this *ad hoc* standard in hopes of laying the initial foundations for a true European digital infrastructure for biomedical research. The success of MIABIS here remains to be seen, but we may not have long to wait: the group will publish an updated version of the model within a year and want to implement it for the new European biobank catalogue as soon as possible (Fig. 7).

## Conclusion

In this article, I have attempted to trace the development of the BBMRI standard information model MIABIS in order to analyse where the values of the political and the technological collide and where European biomedical-policy entanglements (Thévenot 2009) occur. Database integration and information models are not neutral but imbued with politics and conflicts linked to multiple value systems. Information models are among the sites where the ‘biological’ meets the ‘digital’, where biological samples meet information structures, and where national biobanks meet European policy-research interests. Haraway has claimed that ‘the technical and the political are like the abstract and the concrete, the foreground and the background, the text and the context, the subject and the object’ (1997, 10). So it is in the case of MIABIS too. Here, we find interactions in which re-negotiation of many aspects of biomedical research practice takes place and thereby informs the development of large-scale research infrastructures.

The challenges within such projects are not only technical but also philosophical and political. They are philosophical in the sense that ready infrastructures, standards, and digital tools embody translations of ideal principles, central architectures, and key concepts behind the informatics models that form the keystones of digital ontologies. The challenges are political in that deliberate choices are made from among all the alternative conceptual and technical translations presented to the bioinformatics teams working with information models. In the end, the two domains – philosophical and political – are addressed through an entanglement-heavy technical process that most of the time bears little resemblance to what we are accustomed to thinking of as legitimate philosophical or political enquiry focused on EU policies. Thacker elaborates on the many entanglements by writing that ‘agreeing upon what exactly that standard code will be is another matter. There are a range of vested interests (commercial, ideological, institutional, methodological, disciplinary), and the mere decision about standards becomes a discourse on “ontology” in the philosophical sense’ (2004, xxii).

Philosophically speaking, we see how the MIABIS development work dislocates local ‘biobank populations’ from their organismic bodies as biological samples and relocates them to relational databases. Thereby, they become restructured via particular value-imbued information architecture choices. The EU-level database-form integration of national biobanks is aimed at creating a new European population of biomedical bodies that are primarily informational. Databases contain bodies of information (about donor, sample, and study), bodies without biological organs and mediated through an information model that ‘ties together heterogeneous or disparate elements as such: it assures the consolidation of fuzzy aggregates’ (Deleuze & Guattari 2007, 558).

Thus, the network formed through digital tools, the new biobank lexicon, and architectural labours redefines the ‘bio-objects’ held by biobanks in and through the MIABIS model. It is not unifying or totalising in nature. It is instead aggregative and intensifies biological knowledge. This network ties biobanks together in a way that enables new types of bio-objects and bio-objectification practices to emerge by means of the virtual database. In the BBMRI, biological bodies become informational objects composed of digital technologies aimed at fulfilling the promise of European research policy, essentially based on a vision of a distributed infrastructure.

The vision transcends national legislation, policy, and local languages, and it creates a supranational virtual population, whose seat of life is not in the organism but in the database, resisting reduction to existing local, biological, and legal forms of life. This is also a new space where European biomedical bodies – biological samples, study information, medical communities, national co-ordinators, individual biobanks, and policy institutes – are entangled and become enmeshed anew with organ-lacking informational bodies through novel bio-objectification practices.

Instead of political accountability, the bioinformaticians and developers of MIABIS have pragmatic accountability for translating EU research policies into practice. Yet, with this ‘pragmatic accountability’ aimed at a ‘working standard’, the key individuals within the MIABIS working group gain technically mediated power over national biobank definitions. The working, ‘*ad hoc*’ definition of biobanks becomes the *de facto* ‘standard definition’ of a biobank and the bio-objects it contains on the BBMRI-ERIC platform. Any biobank wishing to be included in BBMRI-ERIC must adopt the *ad hoc* definition, regardless of national political or legal debate on the definition of a biobank. Otherwise, it simply cannot be technically listed in the information system keeping track of EU biobanks. This redefinition essentially determines also the range of national biobanks deemed worthy of inclusion in the space of the BBMRI-ERIC platform.

Furthermore, the virtual infrastructure for EU biobanks shifts the boundaries of private and public for individual donors, biological samples, and data as these elements become visible for biomedical researchers and research communities, while it also reorganises their mutual relations in line with the MIABIS ‘attributes’. Although these attributes are deemed ‘meta-information’ about the biobanks, sample collections, and studies, it is this information and the relations between the elements it represents (biobank descriptions, research groups’ and researchers’ descriptions, donors, samples, and information descriptions) that have been at the core of ELSI debates. However, these have less often been seen as something for whose mutual relationships the usual work of bioinformaticians can have wide-ranging legal and political consequences in practice. Kaye (2011) has recently argued that we need to reconsider our traditional way of thinking about governance: it is no longer merely something handled with pen and paper with donors at the beginning of the research. Instead, as the MIABIS case shows well, she argues that in this era of ‘big data’ we ‘should be thinking in terms of data flows and use pathways to enable the sharing and access to data […]. We are moving to a future where biobanks, existing biorepositories and reference databases will be linked and networked for research purposes in ways that has [*sic*] not been possible before’ (*ibid*., 381–382).

Yet our current biobank governance is based on national politics and legal statutes now called into question by global data flows. The idea that all future uses of biobank data are imaginable is untenable, as new uses for data are invented all the time. This is why analysis of standards and infrastructure work is of utmost importance if we are to understand the possibilities and limits of biobanks’ governance and the way in which donors, samples, and data are becoming bio-objectified within large-scale infrastructures today. We hope to be able to learn a new way of thinking about the philosophical and political aspects of standardisation and infrastructure work. This would enable us to keep up with the increasing pace of global integration of biobanks and be better prepared to understand and innovate in the virtualisation of biobank governance.

## Footnotes

1 Available at https://www.bbmriportal.eu/bbmri2.0/jsp/core/login.jsf.

2 See http://bbmri-wiki.wikidot.com/en:dataset.
